# Improved Performance of Graphene in Heat Dissipation when Combined with an Orientated Magnetic Carbon Fiber Skeleton under Low-Temperature Thermal Annealing

**DOI:** 10.3390/ma12060954

**Published:** 2019-03-22

**Authors:** Jing Li, Rubai Lei, Jinfeng Lai, Xuyang Chen, Yang Li

**Affiliations:** 1 School of Chemistry and Chemical Engineering, South China University of Technology, Guangzhou 510641, China; leirubai@foxmail.com (R.L.); laijinfeng666@126.com (J.L.); chenmsy@foxmail.com (X.C.); 2 School of Mechanical & Automotive Engineering, South China University of Technology, Guangzhou 510641, China

**Keywords:** graphene, graphene oxide, carbon fibers, thermal conductivity

## Abstract

The high thermal conductivity and stability, outstanding mechanical properties, and low weight make graphene suitable for many applications in the realm of thermal management, especially in high integration systems. Herein, we report a high-performance, low-temperature reduced graphene oxide/magnetic carbon fiber composite film. Magnetic carbon fibers were prepared using a co-precipitation method, and the graphene oxide solution was prepared using an improved Hummers’ method. The magnetic carbon fibers were orientated by magnetite and immersed in the graphene oxide solution during filtration, followed by annealing at 800 °C. The composite film exhibited improved thermal conductivity (over 600 W/m·K) and mechanical properties (tensile strength of 37.1 MPa and bending cycle of up to 8000). The experimental results illustrate that the graphene in the composite membrane provides heat transfer channels to promote in-plane thermal conductivity, while the magnetic carbon fiber acts as a scaffold to reinforce the mechanical properties and improve the quality of the graphene. Due to the synergistic effect of the graphene and magnetic carbon, this composite has wide potential applications in heat dissipation.

## 1. Introduction

Graphene, a two-dimensional nanomaterial composed of an sp2 hybridized carbon, has attracted tremendous attention due to its high strength, excellent electrical conductivity, and remarkable optical properties [1,2,3]. Owing to these properties, graphene has been used in energy storage batteries [4], supercapacitors [5], and sensors [6]. In thermal management, the integration degree of electronic components continues to increase, and the heat dissipation problem simultaneously increases. Therefore, the demand for highly thermally conductive materials is constantly increasing. Graphene is a highly thermally conductive material, next to diamond [7] and carbon nanotubes [8,9]. The in-plane thermal conductivity of graphene is estimated to be more than 5300 W/m·K [10]. Nevertheless, pristine graphene, formed by mechanical exfoliation [11], liquid exfoliation [12], oxidation–reduction methods [13,14,15], or chemical vapor deposition [16], has no direct commercial applications because the exceptional properties of graphene are typically based on its nanoscale characteristics. Hence, many thermal management products use graphene as a filler [17,18] or graphite paper [19,20,21,22] formed using ultra-high-temperatures and pressure graphitization as a heat transfer medium. However, the preparation of graphite paper is a complex process and involves huge energy consumption in the obtainment of a full graphite structure using different precursors. On the other hand, composites composed of graphene without graphitization for heat transfer also have high thermal conductivities [10], high thermal stabilities [23], outstanding mechanical properties [23,24], and low weights, allowing them to be used in a variety of applications to solve the heat dissipation problems of high integration systems.

The thermal conductivities of carbon materials are closely related to their microstructures. By combining graphene with other materials to form new morphologies, the thermal conductivity of the composites used as thermal interfaces [25,26,27] and lateral heat spreading [28,29,30] materials can be enhanced, synergistic effects can be achieved, new heat conduction channels can be created inside the composites, and the quality of graphene sheets can be improved. However, as most graphene composites are prepared by mixing different components directly to obtain isotropic materials, it is difficult to form periodic morphological structures and control the distribution of graphene. Chaotic internal structures limit the quality and quantity of the directional continuous heat transfer channels, limiting the heat transfer advantages of graphene, especially in a particular direction. Periodic morphology distributions of graphene composites can also enhance the mechanical properties in certain directions.

Carbon fibers are widely used in composite materials as a crucial reinforcement material [31,32]. By choosing different types and sizes of carbon fibers and mixing them with ceramic and polymer substrates directly, the properties of the composite, especially the mechanical properties, can be significantly improved. In this study, a composite material with a periodic distribution of reduced graphene oxide (rGO)/magnetic carbon fiber (MCF) was constructed for heat dissipation applications. Fe_3_O_4_ particles were loaded onto polyacrylonitrile-based carbon fiber (PCF) by co-precipitation [33]. Subsequently, the MCF was dispersed in N-methyl pyrrolidone (NMP), aligned to form a skeleton under the influence of an external magnetic field, and then was deposited on the graphene oxide (GO) using the improved Hummers’ method. Ultimately, we obtained an rGO/MCF membrane by annealing at 800 °C. The quality of the membrane was characterized by Raman spectroscopy, thermogravimetric analysis (TGA), Fourier-transform infrared spectroscopy (FTIR), scanning electron microscopy (SEM), X-ray diffraction (XRD), and transmission electron microscopy (TEM). The rGO/MCF membrane exhibited a thermal conductivity of 600 W/m·K and a tensile strength of 37 MPa. We demonstrated that unidirectional PCF could be formed using a magnetic field. This effectively reduced the number of layers and improved the quality of the graphene, which maximized the thermal conductivity of the graphene filler in the membrane. Furthermore, the PCF improved the mechanical properties of the rGO/MCF membrane by reinforcing it and forming a cladding structure with the rGO. The nanosized Fe_3_O_4_ particles increased the surface roughness between the PCF and graphene, which reduced the slip between the materials and improved the flexibility and tensile strength of the composite film.

## 2. Materials and Methods

### 2.1. Materials

The materials used in this experiment are shown in Table 1.

### 2.2. Preparation of GO and MCF

GO was prepared using the improved Hummers’ method [15]. A 6 mg/mL GO solution was prepared. MCF was obtained using a co-precipitation method. First, we added 2 g PCF to 400 mL acetone and stirred for 48 h, after which the mixture was placed in a water bath and ultrasonicated at 100 W for 1 h. The mixture was vacuum-filtered and dried at 70 °C until a constant weight was achieved. Second, we added dried PCF into a flask followed by 120 mL of nitric acid and 80 mL of sulfuric acid, after which the flask was placed in a water bath at 80 °C for 8 h. The mixture was diluted with deionized water, washed to neutral pH, and dried at 50 °C until a constant weight was achieved. Finally, the dried solids were added to a flask followed by 50 mL deionized water and ferric chloride and ferrous chloride solutions (mFe^3+^:mFe^2+^ = 2:1, mCF–COOH:mFe_3_O_4_ = 2:1). After 24 h, sodium hydroxide was added, and the flask was kept in a water bath at 80 °C until large amounts of precipitates formed. The mixture was left for 1 h, after which it washed to neutral pH and dried at 50 °C to obtain MCF powder.

### 2.3. Preparation of the rGO/MCF Film

**Figure 1:**
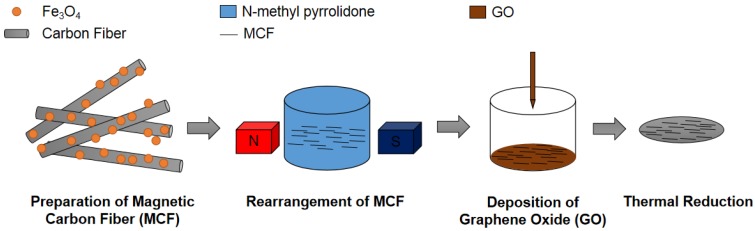
Schematic of the fabrication of the reduced graphene oxide (rGO)/magnetic carbon fiber (MCF) film.

The rGO/MCF film was fabricated using vacuum filtration through a polyvinylidene fluoride microporous membrane (50 mm diameter). After 10 mg of MCF was dispersed in NMP and ultrasonicated at 100 W in a water bath for 10 min, the dispersed solution was poured into a funnel. The funnel was fixed with opposite rubidium magnetite on both sides to create parallel magnetic lines and rearrange the MCF in the process, after which the dispersion was filtered and washed to remove the NMP. Next, 6 mL of GO was added dropwise to a funnel and filtered to form a membrane. The membrane was placed in a vacuum drying oven at 50 °C, after which it was peeled off. The GO/MCF membrane was placed into a mold (smooth graphite plate fixed by heat resisting bolt) and 30 MPa pressure was applied. The mold was placed in a tube furnace in an argon environment that acted as a protective gas, heated at 5 °C/min to 800 °C, and insulated for 2 h to obtain the rGO/ MCF films. The process for the preparation of rGO/MCF films is shown in Figure 1.

### 2.4. Characterization

We used Raman spectroscopy (Renishaw inVia, wavelength 532 nm, measurement range 400–4000 cm^−1^) to examine the structures of graphene and PCF. The thermal stability of the composite film was measured using TGA (TG209F1, Netzsch, heating to 800 °C at a rate of 5 °C/min). The reduction degrees of GO/MCF were measured using FTIR (TENS0R7, Bruker, Karlsruhe, Germany). The morphologies of the MCF and rGO/MCF film were examined using SEM (SU8200, Hitachi, Tokyo, Japan), and the composition was determined by energy disperse spectroscopy (EDS) attached to the SEM. The graphene quality of the rGO/MCF membrane was determined by XRD (X’ Pert Pro, PANalytical, 2 theta range from 5° to 40°) and TEM (JEM-100CX II, JEOL, Tokyo, Japan). The thermal diffusion coefficient of the rGO/MCF was measured using a Laser Flash Apparatus (LFA 447, Netzsch, Selb, Germany, 270 V laser voltage and 0.18 ms pulse width). Due to the anisotropy of rGO/MCF, we used a previously reported method [34] to measure the in-plane thermal diffusivity in directions parallel and perpendicular to the MCF using a sample carrier (diameter 25.4 mm). The in-plane thermal conductivities of the samples at room temperature were calculated using the equation, K = α·ρ·C_p_ (K, thermal conductivity (W/m·K); α, thermal diffusion coefficient (mm^2^/s); ρ, density (g/cm^3^); C_p_, heat capacity (J/g·K)). C_p_ was measured using modulated differential scanning calorimetry (MDSC, DSCQ20, TA instruments, New Castle, PA, USA). A 10-W LED lamp (stable output of 10 W when the input voltage was 24 V) with a copper heat sink on the back was taken. The copper surface was coated with silicone grease, and the rGO, rGO/MCF_⊥_ (where MCF_⊥_ indicates that the MCF in the film was perpendicular to the length of rectangular sample), and rGO/MCF_∥_ (MCF in the film were parallel to the length of the rectangular sample) samples with the same dimensions were placed on the copper heat sink. The surfaces of the samples were coated with black ink to prevent reflection, and the thermal distributions were observed using an infrared thermal imager. The tensile strengths were measured using a universal materials tester (Instron 5967, Boston, MA, USA). The bending cycle of the membrane was determined using a custom device. The membrane was placed on a sample table with two components: One component was fixed and the other moved in a linear reciprocating motion with an adjustable speed driven by a motor to test the membrane at different bending speeds. A photoelectric counter was set on the device to record the cycle numbers.

## 3. Results

**Figure 2:**
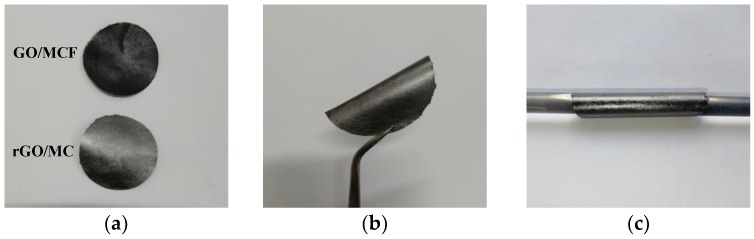
(**a**) Digital images of graphene oxide (GO)/MCF and rGO/MCF films. (**b**,**c**) Digital images of the bending property of the GO/MCF.

Figure 2a shows the photographs of the GO/MCF and rGO/MCF films with diameters of 40 mm. After the addition of the MCF, the surface roughness of the film increased. During the thermal annealing process, GO was reduced to graphene, while the MCF retained its original state and improved the flexibility and bending performance of the composite membrane (Figure 2b). The membrane was able to be wrapped around a 6.5-mm diameter glass rod (Figure 2c).

**Figure 3:**
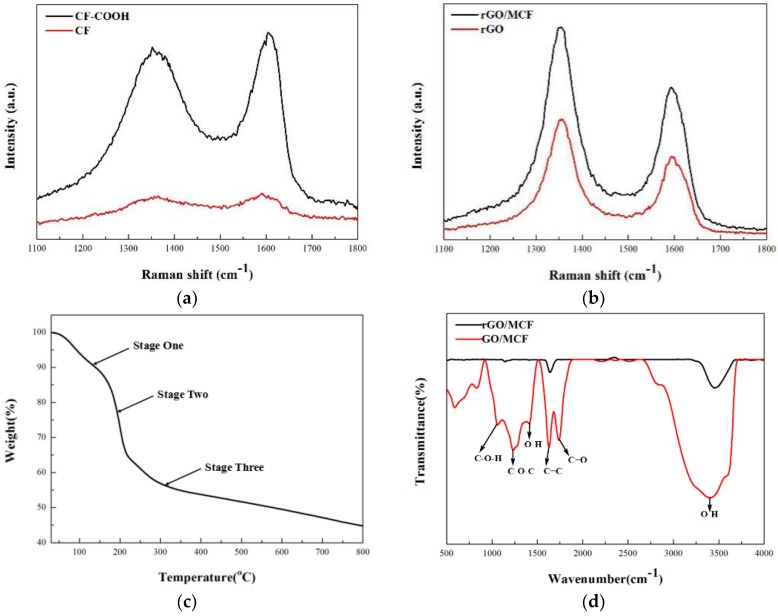
(**a**) Raman spectra of polyacrylonitrile-based carbon fiber (PCF) and carboxylate carbon fiber (CF–COOH). (**b**) Raman spectra of rGO/MCF and rGO. (**c**) Thermalgravimetric analysis (TGA) of the GO/MCF film. (**d**) Fourier-transform infrared spectroscopy (FTIR) spectra of GO/MCF and rGO/MCF.

The textures of the PCF, MCF, rGO, and rGO/MCF films were investigated using Raman spectroscopy. The D and G peaks at 1350 and 1595 cm^−1^, respectively, were the most intense features of the carbon materials. The D peak originated from the breathing modes of the six-membered rings due to the structural defects on the surfaces, and the G peak was typical of sp2 hybridized carbon networks [35]. As shown in Figure 3a, before carboxylation, the half-width of the D and G peaks of the PCF were relatively wide and the spectral lines overlapped. However, after carboxylation, the spectral lines separated and blue-shifted due to the generation of carboxyl groups. Meanwhile, the intensity ratios (I_D_/I_G_) were another important parameter for evaluation of the distance between defects in the graphene [36,37]. As shown in Figure 3b, the removal of functional groups caused defects in the rGO, and, compared to the rGO film, the D and G peaks of rGO/MCF did not deviate significantly. The I_D_/I_G_ ratio decreased from 1.44 for rGO to 1.40 for rGO/MCF, which suggested that the MCF did not change the defect density of the interface in the composite film.

Figure 3c shows the TGA results for GO/MCF from 30 to 800 °C. The thermal reduction process of the composite film was divided into three stages. In the first stage, from 30 to 150 °C, the free water in the composite film was largely removed and the mass fraction decreased by about 12%. In the second stage, from 150 to 200 °C, the bound water in the film converted to free water and was removed. Furthermore, many C–O bonds were broken, releasing CO and CO_2_. Preliminary reduction of the composite film occurred during this stage, and the mass fraction decreased by about 16%. In the third stage, from 200 to 800 °C, the C=O bonds were broken, and the mass fraction of the composite film gradually decreased. The reduction of the GO/MCF film was completed during this stage, and further improvements required higher temperatures. The weight loss of the third stage was approximately 28% [29].

FTIR was used to evaluate the reduction degree of the GO/MCF membrane. As shown in Figure 3d, the peak in the high frequency region around 3400 cm^−1^ was due to stretching vibrations of –OH [30]. The wide spectral peak between 2800 and 3700 cm^−1^ was due to the presence of adsorbed water molecules on the GO. The absorption peak near 1750 cm^−1^ was caused by stretching vibrations of C=O at the edge of the GO, the absorption peak near 1630 cm^−1^ was caused by stretching vibrations of the C=C bond on the surface of the GO. The absorption peaks between 1500 and 1000 cm^−1^ were due to –OH (1410 cm^−1^), C–O–C (1240 cm^−1^), and C–O–H (1080 cm^−1^) vibrations, respectively [38]. After annealing, the C–O–H, C–O–C, C=C, and C=O peaks nearly disappeared. However, the –OH peak due to adsorbed H_2_O was still present.

The morphology of the MCF is shown in Figure S1a, with the elemental distribution obtained by energy disperse spectroscopy (EDS) mapping. The surface of the oxidized PCF contained a large number of carboxyl groups that exchanged cations with Fe^2+^ and Fe^3+^ under an alkaline condition, which generated Fe_3_O_4_ nanoparticles at the positions of carboxyl groups. The magnetization process followed the chemical reaction equation: 2Fe^3+^ + Fe^2+^ + 8OH^−^ = Fe_3_O_4_ + 4H_2_O. The distribution of carbon, oxygen, and iron in the sample are, respectively, shown in Figure S1b–d. After pretreatment, the PCF only contained carbon, and the distribution of carbon was consistent with the contour of PCF. The distribution of oxygen arose mainly from two sources. Before loading with Fe_3_O_4_, the carbon fibers were carboxylated and their surfaces were rich in oxygen functional groups. In addition, Fe_3_O_4_ particles on the surface of PCF also contributed oxygen, and the elemental oxygen was also well-distributed on the surfaces of the PCF. Since iron was only provided by Fe_3_O_4_, which did not completely wrap around the PCF surface, it was distributed sporadically on the PCF surface. The mass fraction of each element of MCF measured by EDS is shown in Table S1. The carbon and oxygen contents were much higher than the iron content, and the oxidation of PCF and the loading of Fe_3_O_4_ occurred on the PCF surface. The average size of Fe_3_O_4_ nanoparticles on the surface of a single MCF is shown in Figure S2 and an average number of them was also calculated.

**Figure 4:**
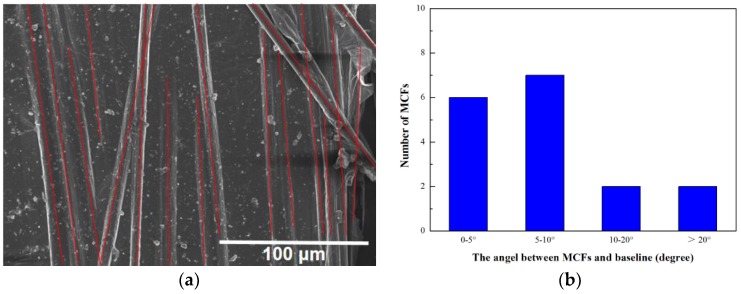

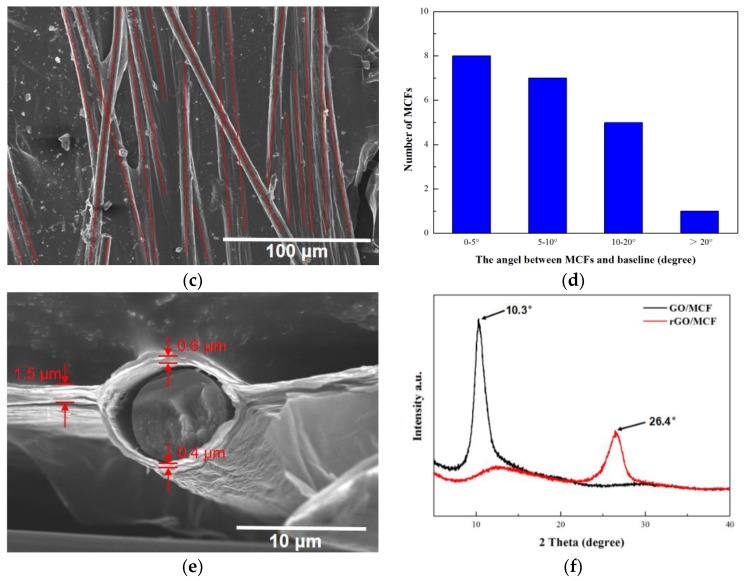
(**a**,**c**) SEM images of the distribution of MCF in rGO/MCF film; (**b**,**d**) distribution of the alignment angles of carbon fibers in different areas of the rGO/MCF membrane; (**e**) cross-section of the rGO/MCF membrane; and (**f**) XRD image of GO/MCF and rGO/MCF.

The morphology of the rGO/MCF is shown in Figure 4a,c. Each Fe_3_O_4_ particle of MCF can be considered a magnetic domain, and when there was no external magnetic field, the magnetic domains were disordered. However, when an external magnetic field was applied, the magnetic domains were arranged according to the magnetic field and the material became ferromagnetic. During the fabrication of the film, the MCF was dispersed in the NMP solution and rearranged in a nearly parallel alignment due to the magnetic field. In the composite film, the MCF formed a parallel skeleton and was covered with graphene. We calculated the alignment distribution of carbon fibers in different areas of the membrane, as shown in Figure 4b,d. In the rGO/MCF film, the angle between most MCF products and the magnetic induction line was less than 20°. A few MCF products contained a small number of magnetic particles, which limited the effect of the external magnetic field, and thus, these MCF products exhibited greater deviation angles from the magnetic induction line direction. Letting 20° be the threshold angle for classifying the MCF as parallel, more than 85% were arranged parallel in the composite membrane. The MCF was not in the same plane as the graphene to form a stacked structure, but was arranged parallel, with different thicknesses throughout the composite film. Furthermore, as shown in the cross-sectional diagram of rGO/MCF in Figure 4e, the MCF inside the membrane separated the graphene sheets, such that the graphene sheet thickness without the MCF coating was 1.5 μm, while the graphene sheets on either side of the MCF was 0.6 and 0.4 μm. When the thickness of the graphene sheets decreased, the probability of out-of-plane scattering in phonon transmission was reduced, which enhanced the in-plane heat transfer of the rGO/MCF composite [39,40]. Simultaneously, the distance between graphene layers also increased. As shown in Figure 4f, the peak at 10.3° was attributed to GO in GO/MCF, while after annealing, the characteristic diffraction peak of GO disappeared and a new peak appeared at 26.4°. The layer distance of the plane was calculated by Bragg’s equation, nλ = 2dsinθ, with a d-spacing of 3.37 Å, which was obviously larger than that of graphite (3.35 Å). This result was also confirmed by the TEM images shown in Figure S3a,b [41].

**Figure 5:**
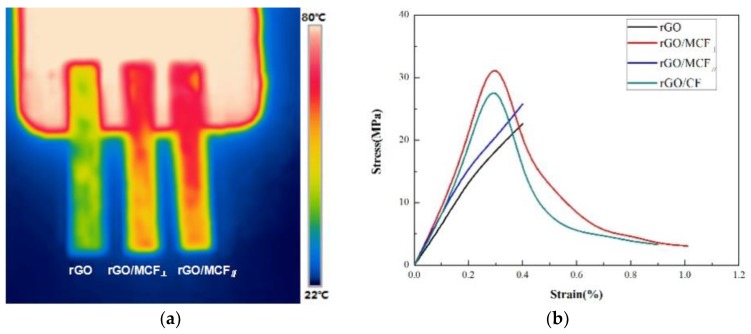
(**a**) Infrared thermal imaging of rGO, rGO/MCF_⊥_, and rGO/MCF_∥_. (**b**) Tensile strengths of rGO, rGO/MCF_⊥_, and rGO/MCF_∥_.

The density of rGO/MCF was 0.51 g/cm^3^, the heat capacity of the rGO/MCF composite film was 0.71 J/(g·K), and the thermal diffusion coefficients of the rGO/MCF_⊥_ and rGO/MCF_∥_ composite films were 1530 and 1650 mm^2^/s, respectively. The thermal conductivities of the rGO (the same preparation method without MCF), rGO/MCF_⊥_, and rGO/MCF_∥_ films were 15, 550, and 600 W/m·K, respectively (the relevant parameters for calculating the thermal conductivity are shown in Table 2). The thermal conductivity of rGO/MCF was significantly higher than that of rGO. The higher thermal conductivity of rGO/MCF indicated that the aligned MCF could separate the graphene sheets and improve their quality from a macroscopic perspective. The thermal conductivity of MCF in the rGO/MCF composite film was only 10 W/m·K at room temperature, which increased the thermal resistance between the rGO and MCF and decreased the thermal conductivity of the composite paper. The parallel MCF in different thicknesses of the film reduced the accumulation of graphene sheets, and the separated high-quality multilayer graphene improved the interface thermal conductivity more efficiently. The phonon leakage of the graphene membrane composed of stacked graphene sheets was an important factor that limited the thermal conductivity. When MCF was added, although its thermal conductivity was lower than rGO, the resulting separation effect could improve the thermal conductivity of rGO/MCF_⊥_ and rGO/MCF_∥_. Although the contact resistance between MCF and rGO had a smaller effect, the contact area of the MCF and rGO of rGO/MCF_⊥_ was higher than rGO/MCF_∥_ in the heat transfer process. The heat dissipation performance of rGO, rGO/MCF_⊥_, and rGO/MCF_∥_ were characterized using an infrared thermal imaging spectrometer, as shown in Figure 5a, where the dimensions of the test samples were 35 mm × 5 mm with thicknesses of 30 μm. When rGO, rGO/MCF_⊥_, and rGO/MCF_∥_ were attached to the copper substrate and the LED reached a stable temperature, rGO/MCF_∥_ had a higher heat-transfer efficiency than rGO and rGO/MCF_⊥_. The temperature distributions of rGO/MCF_⊥_ and rGO/MCF_∥_ were more uniform.

To obtain the mechanical properties of rGO/MCF, the tensile strength and bending cycle were tested. The tensile strengths of rGO, rGO/MCF_⊥_, rGO/MCF_∥_, and rGO/CF_∥_ (Fe_3_O_4_ particles were removed by soaking rGO/MCF in 100 mL of 1:4 hydrochloric acid for 48 h) are shown in Figure 5b. rGO and rGO/MCF_⊥_ had brittle fracture characteristics: The tensile strengths were 22.6 and 25.6 MPa and the Young’s moduli were 5.7 and 6.4 GPa, respectively. The tensile strength of rGO/MCF_⊥_ was higher than that of rGO because the parallel alignment of the MCF skeleton was limited and a few MCF products enhanced the tensile strength in the vertical direction. The tensile strength of rGO/MCF_∥_ had the characteristics of ductile fracture, with a tensile strength of 37.1 MPa and Young’s modulus of over 12.3 GPa. The parallel distribution of MCF directly improved the tensile strength of the composite membrane and provided it with ductile fracture characteristics during stretching, as rGO and MCF must both break for a fracture to occur. The elongation of rGO/MCF was also significantly increased due to the parallel MCF. The nanosized Fe_3_O_4_ particles on the contact surface between MCF and rGO reduced the slip during the tensile process and contributed to the improvement of the tensile strength from 33.2 MPa for rGO/CF_∥_ to 37.1 MPa for rGO/MCF_∥_. The bending test method is shown in Video S1. We cut the test sample into strips of 25 mm × 30 mm and fixed the bending diameter at 5 mm by adjusting the position of the sample on the fixed region of the sample table. No fractures or cracks occurred in the sample after 8000 cycles, and the tensile strength and electric conductivity for 1000–8000 cycles at an interval of 1000 cycles are shown in Figure S4. The sample maintained an excellent thermal conductivity and mechanical properties.

## 4. Conclusions

An rGO/MCF composite with improved heat dissipation was produced using a combination of graphene and a parallel MCF framework under low-temperature thermal annealing. Graphene sheets provided heat channels to provide a high thermal conductivity, while MCF improved the quality of the graphene sheets and the mechanical properties of the composite. The thermal conductivity of rGO/MCF was over 600 W/m·K. Meanwhile, it also had excellent mechanical properties of a 37.1 MPa tensile strength and over 8000 times bending cycle. Hence, the improved thermal conductivity and flexibility of the film can be attributed to a synergetic effect between MCF and rGO. This composite has many potential applications in thermal management.

## Figures and Tables

**Table 1:** The details of the materials used in the experiment.

Name	Origin	Purity (%)
Graphite flakes	Tengshengda Tansu Jixie Co. Ltd., Qingdao, China	99.9
PCF	Anjie Composite Material Co. Ltd., Haining, China	99.9
H_2_SO_4_	Guangzhou Chemical Reagents Factory, Guangzhou, China	A.R.
KMnO_4_	Guangzhou Chemical Reagents Factory, Guangzhou, China	A.R.
H_2_O_2_	Guangzhou Chemical Reagents Factory, Guangzhou, China	30
HCl	Guangzhou Chemical Reagents Factory, Guangzhou, China	G.R.
Acetone	Guangzhou Chemical Reagents Factory, Guangzhou, China	A.R.
HNO_3_	Guangzhou Chemical Reagents Factory, Guangzhou, China	A.R.
FeCl_3_	Guangzhou Chemical Reagents Factory, Guangzhou, China	A.R.
FeCl_2_	Guangzhou Chemical Reagents Factory, Guangzhou, China	A.R.
NMP	Guangzhou Chemical Reagents Factory, Guangzhou, China	99

**Table 2:** Thermal conductivity and relevant parameters of rGO, rGO/MCF_⊥_ and rGO/MCF_∥_.

Name	Density (g/cm^3^)	Heat Capacity (J/(g·K))	Thermal Diffusion Coefficient (mm^2^/s)	Thermal Conductivity (W/m·K)
rGO	0.42	0.64	57.4	15.4 ± 0.8
rGO/MCF_⊥_	0.51	0.71	1658.3	600.4 ± 30.0
rGO/MCF_∥_	0.51	0.71	1532.4	554.9 ± 27.7

## Supplementary Materials

The following are available online at https://www.mdpi.com/1996-1944/12/6/954/s1, Figure S1: (a) SEM imagine of MCF (b) EDS mapping of carbon distribution (c) EDS mapping of oxygen distribution (d) EDS mapping of iron distribution, Figure S2: SEM image of Fe_3_O_4_ nanoparticles on the surface of a single MCF, Figure S3: (a) TEM imagine of rGO in rGO/MCF (b) Layer spacing of rGO/MCF, Figure S4: Thermal conductivity and tensile strength of rGO/MCF_∥_ under different bending cycles, Table S1: Elemental contents of MCF, Video S1: Bending cycle test.
