# Supplementary material for: Improved Performance of Graphene in Heat Dissipation when Combined with an Orientated Magnetic Carbon Fiber Skeleton under Low-Temperature Thermal Annealing
Source: Materials (Basel). 2019 Mar 22;12(6):954. doi: 10.3390/ma12060954 (PMC6471763; doi:10.3390/ma12060954)
Supplement: Supplementary file 1 [file materials-12-00954-s001.zip › materials-469100-supplementary/materials-469100-supplementary.pdf]

materials

Supplementary Information

Improved performance of graphene in heat dissipation when combined with orientated magnetic carbon fiber skeleton under low-temperature thermal annealing

Jing Li [1,*], Rubai Lei [1], Jinfeng Lai [1], Xuyang Chen [1], and Yang Li [2,*]

[1] School of Chemistry and Chemical Engineering, South China University of Technology, Guangzhou, 510641, China; leirubai@foxmail.com (R.L.); laijinfeng666@126.com (J.L.); chenmsy@foxmail.com (X.C.)
[2] School of Mechanical & Automotive Engineering, South China University of Technology, Guangzhou, 510641, China
* Correspondence: ljing@scut.edu.cn (J.L.); meyangli@scut.edu.cn (Y.L.)

Table S1. Elemental contents of MCF.

Element	Mass Content (%)	Atomic Content (%)
C	67.12	74.02
O	30.11	25.48
Fe	2.11	0.50

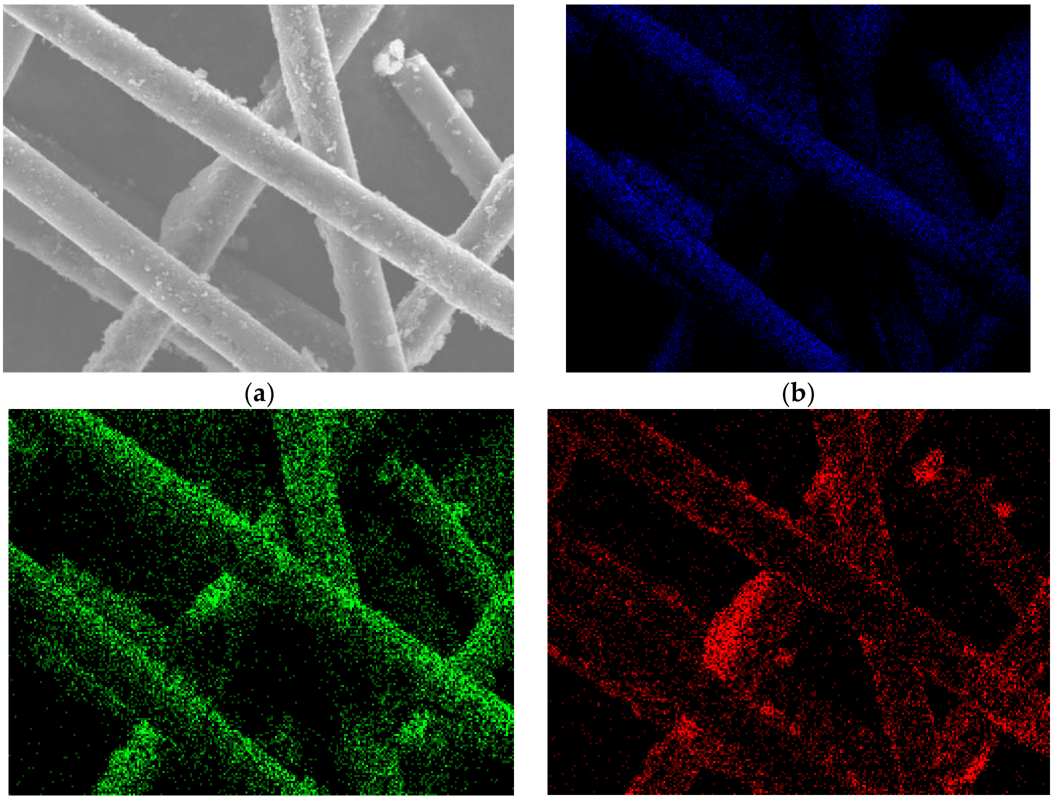

(**a**) (**b**) (**c**) (**d**)

Figure S1. (**a**) SEM image of MCF (**b**) EDS mapping of carbon distribution (**c**) EDS mapping of oxygen distribution (**d**) EDS mapping of iron distribution.

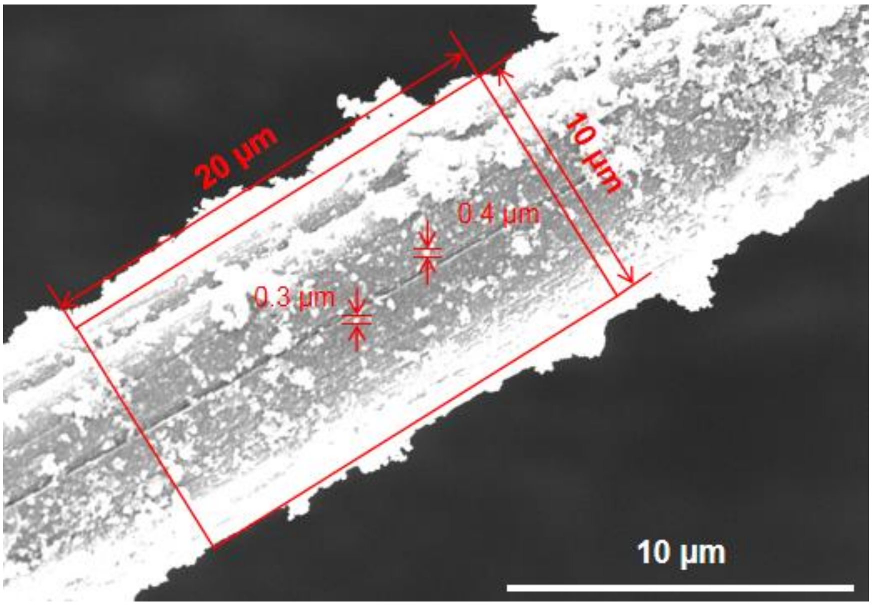

Figure S2. SEM image of Fe3O4 nanoparticles on the surface of a single MCF.

The average size of Fe_3O_4 nanoparticles on the surface of a single MCF is shown in Figure S2., the Fe_3O_4 nanoparticles had obviously agglomeration phenomenon and most of them formed 0.3 to 0.4 μm microspheres even piled up into pieces. The average number of Fe_3O_4 nanoparticles could be calculated roughly. We chose a cylindrical area with a diameter of 10 μm and a height of 20 μm for calculation (The Fe_3O_4 nanoparticles were ignored). The density of CF–COOH was 1.78 g/cm^3, and the volume of the cylindrical region was 1.57×10^{-9} cm^3. The mass content of carbon and oxygen are shown in Table S1, and the average diameter value of Fe_3O_4 nanoparticles was 0.35 μm. The Fe_3O_4 nanoparticles per unit area could be calculated by the following equation:

$$n = (V_{CF\text{-}COOH} \times \rho_{CF\text{-}COOH} \times m_{Fe})/((m_C + m_{O(CF\text{-}COOH)}) \times V_{Fe3O4} \times \rho_{Fe3O4} \times S_{CF\text{-}COOH}) = 8.4 \times 10^7\ cm^{-2} \quad (1)$$

n: the Fe_3O_4 nanoparticles per unit area, $V_{CF\text{-}COOH}$: the volume of chosen CF–COOH cylindrical area, $\rho_{CF\text{-}COOH}$: the density of CF–COOH, m_{Fe}: the ferric mass content of MCF, m_C: the carbon mass content of MCF, $m_{O(CF\text{-}COOH)}$: the oxygen mass content of CF–COOH, V_{Fe3O4}: the volume of a Fe_3O_4 nanoparticle, ρ_{Fe3O4}: the density of a Fe_3O_4 nanoparticle, $S_{CF\text{-}COOH}$: the surface area of chosen CF–COOH cylindrical area

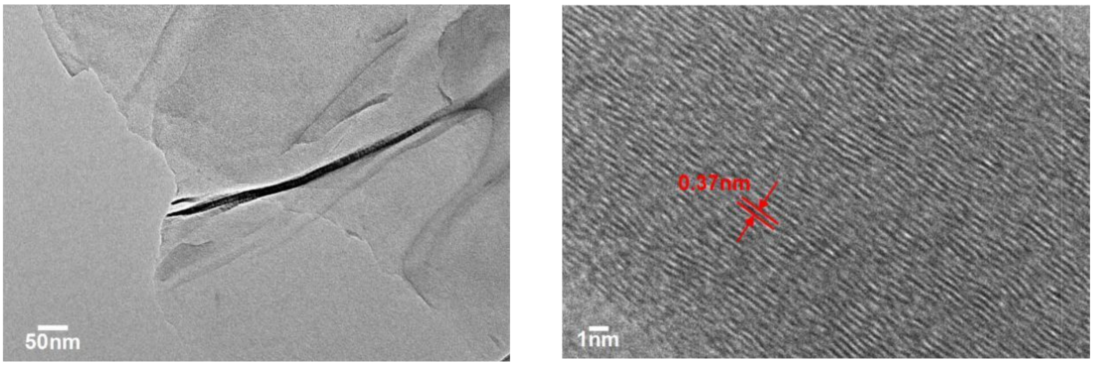

(**a**) (**b**)

Figure S3. (**a**) TEM image of rGO in rGO/MCF film (**b**) Layer spacing of rGO in rGO/MCF.

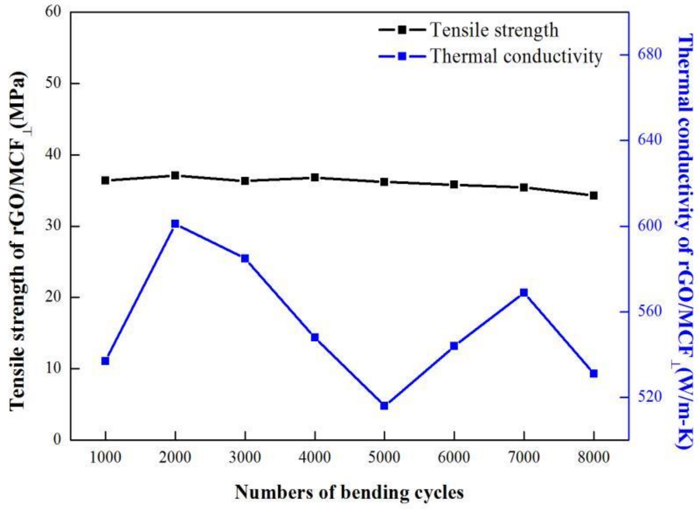

Figure S4. Thermal conductivity and tensile strength of rGO/MCF$_{//}$ under different bending cycles.
